# Fitting the magnetoresponses of the OLED using polaron pair model to obtain spin-pair dynamics and local hyperfine fields

**DOI:** 10.1038/s41598-020-73953-w

**Published:** 2020-10-08

**Authors:** Zhichao Weng, William P. Gillin, Theo Kreouzis

**Affiliations:** Materials Research Institute and School of Physics and Astronomy, Queen Mary University of London, Mile End Road, London, E1 4NS UK

**Keywords:** Spintronics, Organic LEDs, Electronic devices, Organic LEDs

## Abstract

Organic light-emitting diode (OLED) displays a sign reversal magnetic field effect (MFE) when the applied magnetic field range is reduced to the sub-milliTesla range and the Polaron Pair Model has been successful in explaining the ultra-small MFE. Here, we obtained high resolution (~ 1 µT) magnetoconductance (MC) and magnetoelectroluminescence (MEL) of a tris-(8-hydroxyquinoline)aluminium-based (Alq_3_) OLED within the magnetic field range of ± 500 µT with the earth magnetic field components cancelled. A clear “W” shaped MC with a dip position of ± 250 µT and a monotonic MEL were observed. We demonstrate a fitting technique using the polaron pair model to the experimentally obtained MC and MEL. The fitting process extracts physically significant parameters within a working OLED: the local hyperfine fields for electron and hole in Alq_3_: B_hf1_ = (0.63 ± 0.01) mT (electron), B_hf2_ = (0.24 ± 0.01) mT (hole); the separation rates for singlet and triplet polaron pairs: k_S,s_ = (44.59 ± 0.01) MHz, k_T,s_ = (43.97 ± 0.01) MHz, and the recombination rate for singlet polaron pair k_S,r_ = (88 ± 6) MHz. The yielded parameters are highly reproducible across different OLEDs and are in broad agreement with density functional theory (DFT) calculations and reported experimental observations. This demonstrates the feasibility of this fitting technique to approach any working OLED for obtaining significant microscopic parameters.

## Introduction

Since the first report of the magnetoresistance of sexithienyl (T_6_) in a sandwich device structure^1^, there has been ongoing research into organic magnetic field effects (magnetoconductance, MC and magnetoelectroluminescence, MEL) in a range of devices, including spin valves (SVs)^2,3^, organic solar cells (OSCs)^4–6^, organic field-effect transistors (OFETs)^7,8^, and organic light-emitting diodes (OLEDs)^9–14^. In an OLED, the dynamics (dissociation and recombination) of spin pairs (polaron pairs) are involved in both the luminescence and conduction processes. The magnetic field effects (MFEs) in OLEDs were discovered within a small magnetic field range of ± 100 mT and a significant change in optical emission and electrical conductivity were observed^15,16^. Different device structures and organic materials were investigated for their MFEs^17–21^. Normally, the magnetic field range of interest for MFEs research is in the range of tens or hundreds of milliTesla, where a typical monotonic behaviour of MFE (MC or MEL) is present^9,11–13,17,18^. However, when the external magnetic field is reduced to the sub-milliTesla range, a non-monotonic and sign-reversal MFE is also observed^10^. The interaction of an ultra-small magnetic field (e.g. Earth magnetic field ~ 50 µT) with a spin-carrying polaron is significantly smaller (~ 4.5 × 10^5^ times smaller) than the room-temperature thermal energy, and it indicates a thermal nonequilibrium situation is present when the MFE takes place within the sub-milliTesla range, which has attracted considerable attention over the last decade^10,14,22–33^.

Over the tens to hundreds of milliTesla range there have been various theories that have been developed to explain the MFEs^11,13,22,32,34,35^. For example, the bipolaron model^13^, the electron–hole recombination model^34^, the triplet-polaron interaction model^35^, the electron–hole pair model^11^, etc. However, when the external magnetic fields are reduced to the sub-milliTesla range, where the interconversion between singlet and triplet is dominated by the hyperfine interaction, the Polaron Pair Model has been widely used to explain the sign reversal behaviour in the experimentally observed MC^23,24,31,32,36,37^. Investigations and modelling of the polaron pair model have been intensively conducted for the last decade, and the polaron pair model is always capable of generating the same “W” shaped ultra-small field MFE as presented experimentally despite a range of different model conditions^23,24,31,32,36,37^. The polaron pair model takes considerations of different interactions (e.g. hyperfine interaction, Zeeman interaction, exchange interaction, etc.) and dynamics (polaron pairs recombination and dissociation) between polaron pairs or a polaron pair and a nearby hydrogen proton in a molecule. These modelled processes occur in a working OLED, producing the experimentally matched MFE behaviours. However, there is a lack of fitting technique between the polaron pair model and the experimentally obtained MFEs (both MC and MEL) in an OLED, which could allow one to potentially extract significant physical parameters (e.g. polaron pairs recombination/dissociation rates, local hyperfine fields, etc.).

Here we demonstrate a fitting technique using a reduced two proton polaron pair model to our experimentally measured MC and MEL data from the Alq_3_-based OLED. The parameters—polaron pair separation rate, recombination rate, and local hyperfine fields for electron and hole polarons, were extracted from the fitting method, and the measured local hyperfine fields are compared to density functional theory (DFT) calculations from the literature.

## Device, MC and MEL

**Figure 1:**
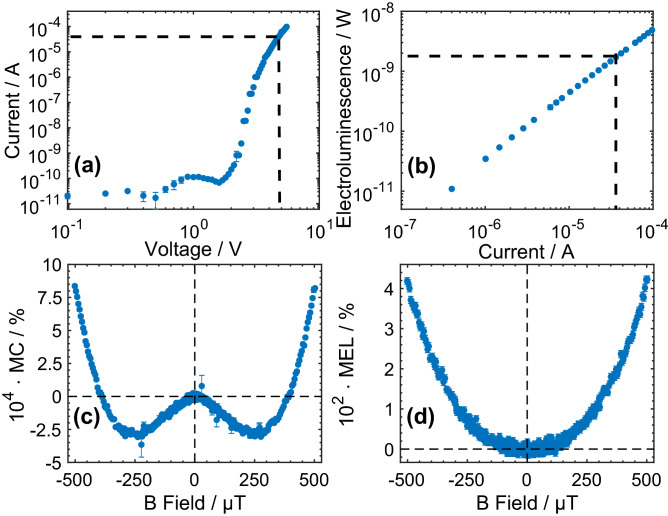
Experimental data of current–voltage (**a**) and Luminescence (**b**) characteristics of a typical device, and Room temperature MC (**c**) and MEL (**d**) under 39 µA drive current, with the Earth’s magnetic field components cancelled. The mean values and error bars (standard error) are calculated over 870 repetitions.

Figure 1a,b show the current–voltage-luminescence characteristics of a typical device and demonstrate that it is operating in a super-linear current–voltage regime with a linear relationship between luminescence and current. These relationships indicate why constant voltage conditions are not suitable for magnetic field effect measurements on such a diode, as the magnetic field dependent device current would simultaneously affect the electroluminescence^38,39^. Figure 1c,d show measured diode MC (note the MC is used to allow for fitting to the theory) and MEL under a constant, 39 µA, drive condition within a range of ± 500 μT, where the MC and MEL can be defined in Eqs. (1) and (2),

1 $$ MC\left( B \right) = \frac{V\left( 0 \right) - V\left( B \right)}{{V\left( B \right)}} \times 100{\text{\% }} $$

2 $$ MEL\left( B \right) = \frac{El\left( B \right) - El\left( 0 \right)}{{l\left( 0 \right)}} \times 100{\text{\% }} $$

where *V(B)*, *V(0)*, *El(B)* and *El(0)* are the measured device voltage and light output under applied magnetic field (*V(B)* and *El(B)*) or zero field (*V(0)* and *El(0)*), respectively.

Figure 1c shows a clear minimum MC at applied fields of B_m_ ~ ± 250 µT. The ultra-small field MC displays typical sign reversal around B_m_^10,14,26,29,33^. At high applied fields the MC follows the well-known monotonic behaviour^2,11,13,15,34^. Noticeably, the MC and MEL behave in a qualitatively different manner as the MEL displays a single monotonic behaviour without the sign reversal seen in the MC. This is because the measured electroluminescence is independent of the MC when operated in the constant current mode which allows us to clearly investigate the separate behaviours of the optical emission (i.e. polaron pairs recombination) and the electroconductivity (i.e. polaron pair dissociation)^39^.

## Two hyperfine fields (two-proton) polaron pair model

The two hyperfine fields (two-proton) polaron pair model has been used in explaining the MFEs in OLEDs^10,23^. The polaron pair model has been successful in qualitatively describing ultra-small magnetic field effects^10,31,33^, and the model has been greatly developed in the past decade^23,24,31,32,36,37^. Here, in order to demonstrate the fitting process of the polaron pair model, a general and simplified two hyperfine field polaron pair model is applied here for the convenience of the fitting, where a reduced stochastic Liouville von Neumann equation is applied, compared to that used in some theoretical works^23,24,31,32,36,37^.

A polaron pair, the precursor of the exciton electron–hole pair, is formed when the distance between the electron and hole is comparable to the Coulomb radius^40^. In the model, each of the polarons in the pair state is coupled to the average hyperfine field it experiences. The difference in the hyperfine fields experienced by the hole and electron can be due to the different spatial distributions of the highest occupied molecular orbital (HOMO) and lowest unoccupied molecular orbital (LUMO) wavefunctions where the hole and electron are located respectively. These two different local hyperfine fields, $B_{hfc1}$ and $B_{hfc2}$, interact with the electron and hole forming the polaron pair respectively. This hyperfine interaction can lift the degeneracy of the polaron pair triplet state even under zero externally applied magnetic field. The external magnetic field can also contribute to the energy splitting of the triplet polaron pair state due to the Zeeman interaction. Hence a simplified Hamiltonian describing the quantum interactions (considering only the Zeeman and hyperfine interactions for the simplicity of the model fitting) of a polaron pair can be expressed by Eq. (3).

3 $$ {\mathcal{H}} = g\mu_{B} B\left( {S_{1z} + S_{2z} } \right) + g\mu_{B} B_{hfc1} {\varvec{S}}_{1} \cdot {\varvec{I}}_{1} + g\mu_{B} B_{hfc2} {\varvec{S}}_{2} \cdot {\varvec{I}}_{2} . $$

where the first term is the Zeeman term due to the external magnetic field, B, for both polarons and the last two terms are the hyperfine interaction terms for the two separate polarons and their corresponding local hyperfine fields. The terms $g$ and $\mu_{B}$ are the electronic g-factor and Bohr magneton respectively and finally, ${\mathbf{S}}$ and ${\mathbf{I}}$ are the spin operators for the polaron and a hydrogen nucleus. The detailed evaluation of the two hyperfine fields (two-proton) polaron pair model carried out in the present work is similar to other literature example^32,41^ in that the electron and the hole experience different local hyperfine fields, but differs in the detail of the Liouville equation and the Hamiltonian (see Supplementary Material).

**Figure 2:**
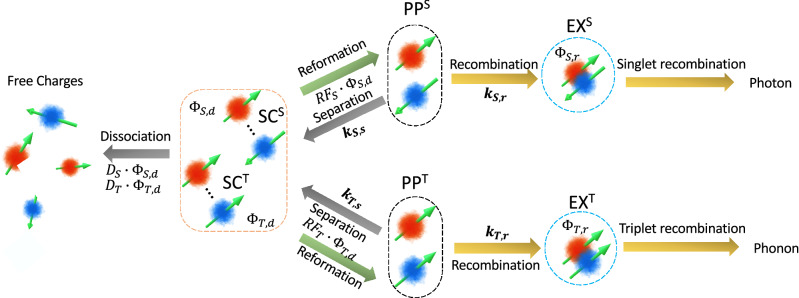
The Schematic for the modelled dynamics for different polaron spin pairs (singlet PP^S^ or triplet PP^T^) in the two hyperfine field (two proton) polaron pair model.

The various processes occurring in the two proton polaron pair model, as used in the present work, are illustrated in Fig. 2. It displays the modelled dynamics for different polaron pairs (singlet PP^S^ or triplet PP^T^), and the hole and the electron polarons forming the pair experience different local hyperfine fields, $B_{hfc1}$ and $B_{hfc2}$ respectively. Each polaron pair (either singlet or triplet) undergoes two dynamic decay processes over time. The first is the recombination to form a tightly bound exciton state (EX^S^ or EX^T^) and the second is the separation into separate charges (SC^S^ or SC^T^). The separate charges can then evolve by either reforming the original polaron pair or fully dissociating into free charge carriers. In the case of the recombination pathway, the singlet and triplet polaron pairs form tightly bound excitons with rates $k_{S,r}$ and $k_{T,r}$ respectively, whereas in the separation pathway they separate into loosely bound charge pairs with rates $k_{S,s}$ and $k_{T,s}$ respectively. The separated charge states, SC^S^ and SC^T^, either fully dissociate with equal probabilities $D_{S}$ = $D_{T}$ = $D$, or reform the original singlet and triplet polaron pair states with equal probabilities $RF_{S}$ = $RF_{T}$ = $RF$. In contrast to these states, the exciton states (EX^S^ or EX^T^) are always considered to undergo recombination.

The initial polaron pair states, PP^S^ or PP^T^, undergo oscillations in their nature under the influence of the different local hyperfine fields experienced by the holes and electrons forming them as well as any external magnetic field presents. By considering the temporal evolution of the polaron pairs, including the decay processes of the initial polaron states, we can evaluate the individual yield for each process. The yields are ${\Phi }_{S,r}$, ${\Phi }_{T,r}$, ${\Phi }_{S,s}$ and ${\Phi }_{T,s}$ for the recombination and separation of initial singlet and triplet state respectively.

The singlet exciton yield, ${\Phi }_{S,r}$ contributes to the device luminescence by photon emission, whereas the separated charge yields, ${\Phi }_{S,s}$ and ${\Phi }_{T,s}$, contribute to the current flowing through the device via the dissociation into free charges. The yields ${\Phi }_{S,r}$, ${\Phi }_{S,s}$ and ${\Phi }_{T,s}$ depend not only on the two hyperfine fields experienced by the electron and hole of the original polaron pairs, but also on any externally applied magnetic field. Hence, the dynamics described in Fig. 2 can give rise to both MEL and MC. We note that the recombination of the triplet exciton yield, ${\Phi }_{T,r}$, does not contribute to any experimentally observed quantity in this model, hence the recombination of the triplet polaron pair process is not included in our simplified two hyperfine field polaron pair model.

In Fig. 2, the separate magnetic field dependent decay process yields occurring in a device are indicated by the use of the common notation$, {\Phi }$, denoting yield. For the simplified two hyperfine field model used in fitting the device results in this work, the separate yields are evaluated using Eqs. (4)–(6).

4 $$ {\Phi }_{S,r} = k_{S,r} \cdot \mathop \smallint \limits_{0}^{\infty } \rho_{S} \left( t \right)e^{{ - k_{S,r} \cdot t}} dt $$

5 $$ {\Phi }_{S,s} = k_{S,s} \cdot \mathop \smallint \limits_{0}^{\infty } \rho_{S} \left( t \right)e^{{ - k_{S,s} \cdot t}} dt $$

6 $$ {\Phi }_{T,s} = k_{T,s} \cdot \mathop \smallint \limits_{0}^{\infty } \rho_{T} \left( t \right)e^{{ - k_{T,s} \cdot t}} dt $$

where $t$ denotes time and the quantities $\rho_{S} \left( t \right)$ and $\rho_{T} \left( t \right)$ are the time dependent spin densities of the singlet and triplet polaron pair states respectively. Theoretically, spin-selective polaron pair interactions can be calculated explicitly for different dynamics (recombination and dissociation) using a more comprehensive and well developed Liouville equation as defined by many theoretical works^23,24,31,32,36,37^. This more rigorous approach, however, falls outside the scope of the present work of model fitting and we use the simple approach where the recombination and dissociation dynamics of singlet polaron pair states are simultaneous and independent processes as shown in Eqs. (4) and (5). We note that the approach used allows the temporal evolution of initial singlet and triplet states to occur under the influence of an external magnetic field into the intermediate excitonic (EX) and separated charge (SC) states, returning the relevant yields, before any dissociation or decay processes have taken place. Thus, the use of a reduced Liouville equation in the evaluation of the yields (see Supplementary Material) is justified, since our approach conserves total particle numbers and energy. After the yields are evaluated, both dissociation and reformation are defined using their respective probabilities ($D$ or $RF$). This reduced model, for the simplicity of the model fitting, differs from the comprehensive theoretical approach taken by other authors, where an expanded Liouville equation or Hamiltonian is used to allow for the non-conservation of total energy or particles^23,24,42^. In a similar manner to the literature, we also evaluate the different relative yields resulting from an initial population of singlet or triplet state polaron pairs over a single time interval and generalize to the steady-state applicable to a device under study.

Our polaron pair model fitting technique includes a global fitting of corresponding MC and MEL results obtained from a given device using a single set of fitting parameters, namely: $B_{hf1}$, $B_{hf2}$, $k_{S,s}$, $k_{T,s}$ and $k_{S,r}$. The MC and MEL are formulated using the first principle definitions as given in Eqs. (7) and (8).

$$ MC\left( B \right) = \frac{{\left( {D_{s} {\Phi }_{S,s} \left( {B \ne 0} \right) + D_{T} {\Phi }_{T,s} \left( {B \ne 0} \right)} \right) - \left( {D_{s} {\Phi }_{S,s} \left( {B = 0} \right) + D_{T} {\Phi }_{T,s} \left( {B = 0} \right)} \right) }}{{D_{s} {\Phi }_{S,s} \left( {B = 0} \right) + D_{T} {\Phi }_{T,s} \left( {B = 0} \right)}} $$

where $$D_{S} = D_{T} = D$$, yielding

7 $$ MC\left( B \right) = \frac{{{\Phi }_{S,s} \left( {B \ne 0} \right) + {\Phi }_{T,s} \left( {B \ne 0} \right) }}{{{\Phi }_{S,s} \left( {B = 0} \right) + {\Phi }_{T,s} \left( {B = 0} \right)}} - 1 $$

8 $$ MEL\left( B \right) = \frac{{{\Phi }_{S,r} \left( {B \ne 0} \right) - {\Phi }_{S,r} \left( {B = 0} \right) }}{{{\Phi }_{S,r} \left( {B = 0} \right)}} $$

The difference between the definition of the MC in Eq. (7) and the approach used in literature is the ambiguous weight factor $\delta_{TS}$, which is used to account for a notional difference in the dissociation rate for singlets and triplets^32^; in our approach no such factor is necessary, and the MC naturally follows the different separation yields for singlets and triplets which share the same probability of dissociation after separation. The MEL expression in Eq. (8) is straightforward as the experimentally observed electroluminescence is only related to the population of the singlet excitons. Hence, we can directly obtain the rate constant for singlet polaron pair recombination, $k_{S,r}$ via model fitting. We note that the actual radiative recombination yield for the singlet is irrelevant in the MEL as it would be equally applied to each term and cancel out.

## Model fitting and analysis

The data-model optimization and fitting process are realized by minimizing a global reduced $\chi^{2}$ as discussed in Supplementary Material.

**Figure 3:**
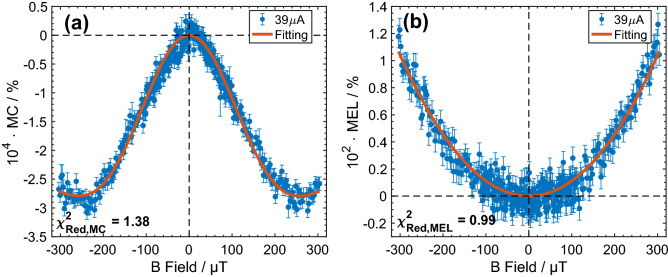
Experimental data and model fitting. Experimental MC (**a**), and MEL (**b**), obtained from an ITO/NPB (50 nm)/Alq_3_ (50 nm)/LiF (1.5 nm)/Al (100 nm) device under 39µA constant drive current with the Earth magnetic field cancelled. The red solid lines are the MC and MEL obtained by global fitting of the two local hyperfine field model. The mean values and error bars (standard error) are calculated over 870 repetitions.

The fitting procedure has been carried out on experimentally obtained MC and MEL as shown in Fig. 3. The parameters returned are: $$B_{hf1} = \left( {0.63 \pm 0.01} \right)$$ mT, $$B_{hf2} = \left( {0.24 \pm 0.01} \right)$$ mT, $$k_{S,s} = \left( {44.59 \pm 0.01} \right) $$ MHz, $$k_{T,s} = \left( {43.97 \pm 0.01} \right) $$ MHz and $$k_{S,r} = \left( {88 \pm 6} \right) $$ MHz with $$\chi_{red,MC}^{2} = 1.38$$ and $$\chi_{red,MEL}^{2} = 0.99$$. We note that these parameters are reproducible across different devices and are independent of drive current (see Supplementary Material). The rate constants are in agreement with the approximate lifetime of the polaron pair (~ 10 ns equivalent to a decay rate of ~ 100 MHz)^43^. The obtained rate constant for singlet polaron-pair recombination ($$k_{S,r} = $$ 88 MHz) is higher than the corresponding rate constant for separation ($$k_{S,s} =$$ 44.6 MHz) and this accounts for the significantly larger magnitude of the MEL compared to the MC. However, it is interesting that the difference in rate constants between the recombination and separation is only a factor of 2, demonstrating that when polaron pairs are first formed a significant proportion will undergo separation.

**Figure 4:**
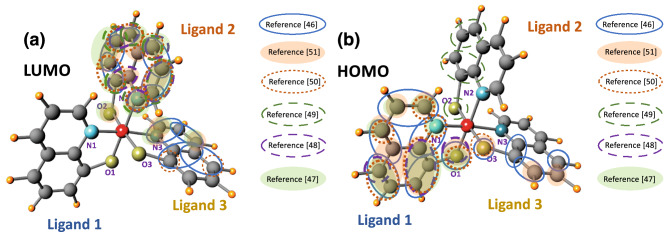
The Schematic of the 3 hydroxyquinoline ligands in an Alq_3_ molecule showing: The spatial distribution of (**a**) the LUMO and (**b**) the HOMO wavefunctions. The HOMO and LUMO molecular orbital positions correspond primarily to ligand 1 and ligand 2, respectively. The different coloured lines circling individual atoms indicate the literature source of the atoms corresponding to the relevant HOMO or LUMO spatial distribution^46–51^.

We have obtained significantly different values for the two local hyperfine fields, $B_{hf1} = 0.63$ mT and $B_{hf2} = 0.24$ mT experienced by the electron and hole forming the polaron pair. This notable difference between the electron and the hole local hyperfine fields was also observed experimentally from literature^32,41,44,45^. The polarons are experiencing different local hyperfine fields despite both being localized on an Alq_3_ molecule. This is accounted for by the fact that the electron resides in the LUMO of the Alq_3_ molecule whereas the hole is in the HOMO. The HOMO and LUMO wave functions (orbitals) are spatially separated on the molecule and the charges residing within them therefore experience different local hyperfine fields. There are many density functional theory (DFT) based calculations in the literature which provide information on both the spatial extent of the HOMO and LUMO wave functions as well as the local hyperfine fields due to different atoms in Alq_3_^46–51^. Theoretically, the average local hyperfine fields experienced by an electron or hole forming a polaron in Alq_3_ can be approximated using DFT values available from the literature^24,52^. The method itself is based on a weighted sum of the hyperfine field due to individual atoms and the spin quantum number of the atoms with the sum carried out over all atoms corresponding to the spatial extent of the HOMO or LUMO wavefunction. The details of the method used to obtain the average local hyperfine fields can be found in Supplementary Material. Using the literature values of the hyperfine component of different atoms in Alq_3_ we have estimated the local hyperfine fields of $\left\langle {B_{hf - LUMO} } \right\rangle \approx$ 2.13 mT and $\left\langle {B_{hf - HOMO} } \right\rangle \approx$ 0.36 mT, meaning that the electron and hole experience completely different hyperfine fields^53^. The electron is immersed in a relatively large hyperfine field environment, whilst the hole only senses a relatively small hyperfine field in an Alq_3_ molecule. According to the literature, the electron and hole reside primarily in ligand 2 and ligand 1 of an Alq_3_ molecule respectively, as shown in Fig. 4a,b^46–51^, and the intensity for the local hyperfine interaction is inverse proportional to the size of the localization of the polaron^44^. From Fig. 4a,b, the electron residing in the LUMO position in Alq_3_ molecule has smaller localization areas compared to that of the hole (HOMO’s position in an Alq_3_ molecule is possible over all three ligands according to different theoretical works^46–51^), indicating a larger electron local hyperfine field and a smaller hole local hyperfine field in an Alq_3_ molecule. Noticeably, the two hyperfine fields obtained from the MC and MEL data fitting, $B_{hf1} = \left( {0.63 \pm 0.01} \right)$ mT and $B_{hf2} = \left( {0.24 \pm 0.01} \right)$ mT, are significantly different from each other, suggesting that the hyperfine field components obtained by fitting can not only represent the local hyperfine fields for an electron or hole in an Alq_3_ molecule, but also estimating that the larger hyperfine field component ($B_{hf1} = \left( {0.63 \pm 0.01} \right)$ mT) corresponds to electron’s while the smaller component ($B_{hf2} = \left( {0.24 \pm 0.01} \right)$ mT) relates to the hole’s.

However, there is a discrepancy between our estimated values ($\left\langle {B_{hf - LUMO} } \right\rangle \approx$ 2.13 mT and $\left\langle {B_{hf - HOMO} } \right\rangle \approx$ 0.36 mT) from DFT and our extracted values through data fitting ($B_{hf1} = \left( {0.63 \pm 0.01} \right)$ mT and $B_{hf2} = \left( {0.24 \pm 0.01} \right){ }$ mT). The difference primarily comes from the difficulty in obtaining reliable values from the DFT data presented in the literature^46–51^, where different DFT approaches give a range of values for the distribution of the LUMO and HOMO across each atom. This is further exacerbated by the way the DFT results are presented by different authors which makes precise determination of the weighting in each paper difficult. We have therefore had to determine an “average” distribution from different papers. This is the major source of discrepancy between the DFT and our results. This approach is fully discussed in the Supplementary Material. However, it is important to note that our simulation results demonstrate a significant difference between the two fitted local hyperfine field values corresponding to the electron and hole polarons and that this difference is also seen in the estimated local hyperfine fields from DFT simulations, even though there are discrepancies in the absolute value of the difference. Furthermore, from our simulation (see Supplementary Material) we can see that the DFT estimated local hyperfine fields are also able to generate the functional “W” shape MC with appropriate dynamics rates ($k_{S,s} , k_{T,s} ,k_{S,r}$), indicating the validity of our estimated values. However, a comprehensive DFT simulation on the local hyperfine fields in an Alq_3_ molecule falls out of the scope of this work.

The fitting results additionally indicate that the separation rates ($k_{S,s} \sim 45$ MHz or $k_{T,s} \sim 44$ MHz) are much smaller than the singlet recombination rate ($k_{S,r} \sim 88$ MHz), which indicates that polaron recombination dominates, although a significant number of polaron pairs do separate after formation. Reference^36^ demonstrates that the decay rate (recombination or separation) ratio to the hyperfine Larmor frequency should be small to yield a “W” shaped magnetic field effect. In our model, we found this threshold rate ratio is approximately 0.57, above which the functional “W” shaped MC starts to disappear. Further details regarding this simulation can be found in Supplementary Material. From the fitting results, the recombination rate to hyperfine Larmor frequency ratio ($k_{S,r} /\omega_{hfc}$ ~ 0.79) is larger than in the case of the separation rate ($k_{S or T,s} /\omega_{hfc}$ ~ 0.4). This is consistent with the absence of any functional “W” shape in measured MEL, whereas the smaller separation rate to Larmor frequency ratio is consistent with the “W” shape observed in MC. Considering the magnitudes of the measured MC and MEL (Fig. 3) it is clear that the MEL is two orders of magnitude greater than the MC in the same device. This is consistent with the emissive decay probability of a recombined polaron pair (EX^S^) being unity, in contrast to the dissociation probability of a separated polaron pair (SC^S^ or SC^T^) which is significantly smaller than unity as a result of the non-negligible reformation probability.

The electron local hyperfine field of Alq_3_ at room temperature is too small for generating an ultra-small magnetic field feature (“W” form). According to polaron pair model^36^, the ultra-small magnetic field effect will manifest itself when the rate is small compared to the hyperfine Larmor frequency. In simulations (see Supplementary Material) we find that ultra-small magnetic field MEL feature will start to show when the ratio of singlet recombination rate over the Larmor frequency is smaller than 0.51 in Alq_3_ molecule. Given the measured singlet recombination rate ($k_{S,r} = 88$ MHz) this indicates that the electron hyperfine field would need to be larger than 0.98 mT. However, the ratio in our fitted result is 0.79, which is significantly greater than the threshold ratio value, and the electron hyperfine field is 0.63 mT, which is smaller than 0.98 mT, hence no “W” feature should be observed. In the literature^10,30^, there are a several papers which have observed the “W” shaped MEL in some OLEDs, including Alq_3_ based devices, at room temperature. However, in these papers the MC and MEL were measured in constant voltage mode and the device current was varying with applied field. As device current is linearly related to the EL of the OLED, when the device current is showing a “W” shaped behaviour, so will the EL.

The fitting technique in this work using two hyperfine field polaron pair model can extract physically significant parameters (local hyperfine fields for electron and hole, and dissociation and recombination rates for polaron pair) for a working OLED, however, for the simplicity of the fitting, only a simplified two hyperfine field polaron pair model is applied here. Further developments are required for the current simplified theoretical model for higher accuracy of the yielding parameters, for example, the inclusions of other possible quantum interactions (exchange interaction, triplet-polaron quenching, etc.), a more comprehensive stochastic Liouville equation as modelled from literature^23,24,31,32,36,37^, and an alternative modelling for steady-state charge pair dynamics^54^, etc.

In conclusion, we demonstrate the different functional shapes of the experimentally measured MC and MEL of the Alq_3_-based OLED within the ultra-small magnetic field range (± 500 µT) under the constant current mode. The MC is displaying a characteristic “W” shaped form (dip position at ± 250 µT) while the MEL behaves in a monotonic manner, indicating two independent optical and electrical processes under the constant current mode^39^. A simplified two hyperfine field polaron pair model has been fitted to the experimentally obtained MC and MEL of the Alq_3_-based OLED. The fitting was carried out globally on both the MC and MEL data, and it yields 5 physically significant parameters: the local hyperfine fields for electron and hole in Alq_3_: $B_{hf1} = \left( {0.63 \pm 0.01} \right)$ mT (electron), $B_{hf2} = \left( {0.24 \pm 0.01} \right)$ mT (hole); the separation rates for singlet and triplet polaron pairs: $k_{S,s} = \left( {44.59 \pm 0.01} \right)$ MHz, $k_{T,s} = \left( {43.97 \pm 0.01} \right)$ MHz, and the recombination rate for singlet polaron pair $k_{S,r} = \left( {88 \pm 6} \right)$ MHz. The yielded parameters are highly reproducible across different devices and show the same broad difference in electron and hole hyperfine environment as theoretical DFT works and experimental observations from literature^46–51^. The fitting technique reported in this work is shown to be applicable in extraction of physically significant microscopic parameters (e.g. the local hyperfine fields and polaron pair dynamics rates) of any working OLED.

## Methods

### Device fabrication

The device used in this work consists of 5 layers: indium tin oxide (ITO) as the anode, *N*,*N*′-Di(1-naphthyl)-*N*,*N*′-diphenyl-(1,1′-biphenyl)-4,4′-diamine (NPB) as the hole transport layer (HTL), tris-(8-hydroxyquinoline)aluminium (Alq_3_) as the electron transport and emissive layer (ETL/EL), LiF as the electron injection layer (EIL) and aluminium as the cathode: ITO/NPB (50 nm)/Alq_3_ (50 nm)/LiF (1.5 nm)/Al (100 nm) with a device area of 4 mm^2^. NPB and Alq_3_ were purchased from Sigma-Aldrich Inc. and purified twice using train sublimation before use. Pre-patterned ITO glass substrate was thoroughly cleaned before any material deposition. The device processing parameters using organic deposition system are: 10^−7^ mbar base pressure, ~ 0.2 nm s^−1^ NPB and Alq_3_ deposition rates, 0.02 nm s^−1^ LiF deposition rate, 0.06 nm s^−1^ for the initial 10 nm and 0.5 nm s^−1^ thereafter for Al deposition.

### Magnetic field effect measurements and data analysis

The MC measurements were carried out with the Earth’s magnetic field components cancelled using a 3-D Helmholtz coil system, which was driven by a Keithley 2400 SourceMeter. The device drive current was applied by an Agilent B2902A source-measure unit and the device voltage was obtained using a Keithley 4200 semiconductor characterization system. A LakeShore 475 DSP Gaussmeter was applied to the magnetic field measurements which were controlled by a custom-written software. The gaussmeter recorded the actual B fields at each data point and provided data for the plots, where the B-field step sizes are ~ 1 µT, 2.5 µT and 5 µT in the B field regimes of ± 100 µT, ± (100–300) µT and ± (300–500) µT, respectively. The device voltages were measured under different externally applied magnetic fields under a constant device drive current (39 µA). The mean values and error bars of the data were calculated using algorithmic mean over 870 repetitions of measurements and standard errors, yielding high data sensitivity of 10^−7^. The device drift during measurements was inevitable, and it was eliminated after a quantitative analysis by averaging the two zero field readings (before and after a non-zero field reading). Additionally, the light output of the OLED was recorded using a photodetector and an optical power meter (Newport 1830-C). The photodetector is coupled to the OLED using an optical fibre to prevent stray magnetic fields from steel screws inside the photodetector affecting the sample.

All measurements were taken at room temperature with the diode under vacuum (10^–5^ mbar) using a specially designed sample holder with no ferromagnetic components. We note that no measurable device degradation was observed during long device operation time (see Supplementary Material).

## Supplementary information

Supplementary Information.

## Data Availability

The datasets generated and/or analysed during the current study are available from the corresponding author on reasonable request.
