# Supplementary material for: Fitting the magnetoresponses of the OLED using polaron pair model to obtain spin-pair dynamics and local hyperfine fields
Source: Sci Rep. 2020 Oct 8;10:16806. doi: 10.1038/s41598-020-73953-w (PMC7544898; doi:10.1038/s41598-020-73953-w)
Supplement: Supplementary file 1 — Supplementary Information. [file 41598_2020_73953_MOESM1_ESM.pdf]

Supplementary Material

Fitting the Magnetoresponses of the OLED using Polaron Pair Model to Obtain Spin-Pair Dynamics and Local Hyperfine Fields

Zhichao Weng*, William P. Gillin and Theo Kreouzis
Materials Research Institute and School of Physics and Astronomy, Queen Mary University of London, Mile End Road, E1 4NS, London, United Kingdom
Email: z.weng@qmul.ac.uk

S1. Two proton polaron pair modelling

A reduced energy-conserved stochastic Liouville von Neumann is applied as shown in equation (SE1)

$$i\hbar\left(\frac{\partial\sigma(t)}{\partial t}\right) = [\mathbf{H}, \sigma] \qquad \text{SE1}$$

The solution to equation (SE1) can be obtained[36, S1] as in equation (SE2)

$$\sigma(t) = e^{-iHt}\sigma(0)e^{iHt} \qquad \text{SE2}$$

where the initial assumption of the formation of singlet polaron pair is made (this is arbitrary and the same physics can be obtained by assuming initial triplet formation) with initial singlet density, σ(0)[36]. $\mathbf{H}$ is the spin Hamiltonian consisting of different interactions among polaron pairs and the external magnetic field. Those interactions include Zeeman interaction between each polaron and the external magnetic field, the hyperfine interaction between the polaron and its surrounding hydrogen nuclei, the dipolar interactions and exchange interactions between the spins of each polaron, etc. However, a reduced Hamiltonian containing only the Zeeman and hyperfine interactions is applied for simplicity of calculation. For this work, the hyperfine interaction has two protons coupling to each of the polarons as shown in equations (SE3a), (SE3b) and (SE3c)

$$H = H_{Zeeman} + H_{Hyperfine} \qquad \text{SE3a}$$

$$H_{Zeeman} = g\mu_B B \cdot (S_{1z} + S_{2z}) \quad \text{SE3b}$$

$$H_{Hyperfine} = g\mu_B \cdot [B_{hfc1} \cdot (\mathbf{S}_1 \cdot \mathbf{I}_1) + B_{hfc2} \cdot (\mathbf{S}_2 \cdot \mathbf{I}_2)] \quad \text{SE3c}$$

Where g is the g-factor that is approximately equal to 2.002, μ_B is the Bohr magneton 5.788×10^{-5} eV·T^{-1}, B is the applied magnetic field, S_{1z} and S_{2z} are the z components of the spin operators for the two polarons and B_{hfc1}, B_{hfc2} are the local hyperfine fields due to two protons. $\mathbf{S}_1$ and $\mathbf{S}_2$ are the spin operators including all components for both polarons and $\mathbf{I}_1$ and $\mathbf{I}_2$ are the spin operators for the hydrogen nuclei. The terms $\mathbf{S}_1 \cdot \mathbf{I}_1$ and $\mathbf{S}_2 \cdot \mathbf{I}_2$ can be defined in equations SE4 (a) and (b)

$$\mathbf{S}_1 \cdot \mathbf{I}_1 = \mathbf{S}_{1x} \cdot \mathbf{I}_{1x} + \mathbf{S}_{1y} \cdot \mathbf{I}_{1y} + \mathbf{S}_{1z} \cdot \mathbf{I}_{1z} \quad \text{SE4a}$$

$$\mathbf{S}_2 \cdot \mathbf{I}_2 = \mathbf{S}_{2x} \cdot \mathbf{I}_{2x} + \mathbf{S}_{2y} \cdot \mathbf{I}_{2y} + \mathbf{S}_{2z} \cdot \mathbf{I}_{2z} \quad \text{SE4b}$$

Likewise, $\mathbf{S}_1$, $\mathbf{I}_1$, $\mathbf{S}_2$ and $\mathbf{I}_2$ with different x, y and z subscripts correspond to the Pauli matrices for the polarons and the hydrogen nuclei respectively.

Figure S1 is a schematic of the two-proton hyperfine coupling situation, where each polaron is coupled to its neighbouring hyperfine field.

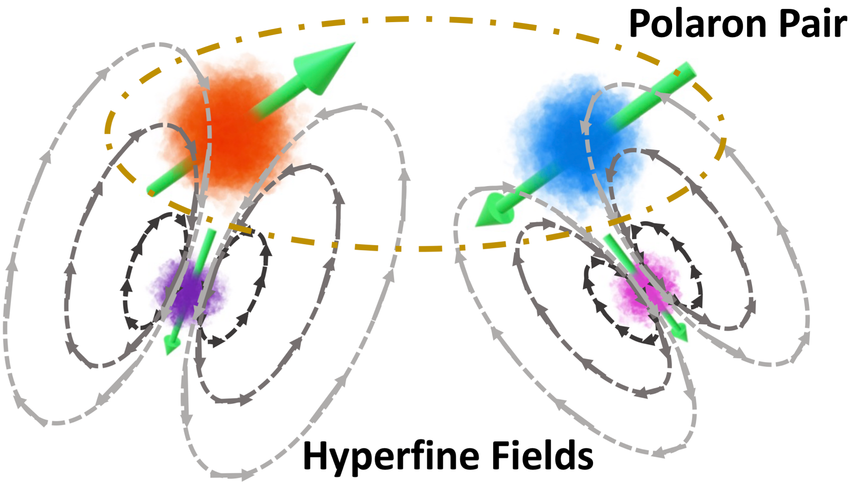

Figure S1: The illustration of the so-called two-proton hyperfine coupling

The singlet fraction, ρ_s, is calculated via the trace of the singlet projection operator, P_s, operating on $\sigma(t)$ as shown in equation SE5:

$$\rho_s = \mathrm{Tr}[P_s \sigma(t)] \qquad \text{SE5}$$

The singlet-triplet transformation basis is necessary for later calculation basis transformation as given in Equation SE6

$$P_2 = \begin{pmatrix} 1 & 0 & 0 & 0 \\ 0 & {}^{1}/_{\sqrt{2}} & {}^{1}/_{\sqrt{2}} & 0 \\ 0 & -{}^{1}/_{\sqrt{2}} & {}^{1}/_{\sqrt{2}} & 0 \\ 0 & 0 & 0 & 1 \end{pmatrix} \qquad \text{SE6}$$

Where the second column represents singlet component while the other three columns represent three different triplet components. For consistency of the quantum calculation, all the spin components should stay in the same singlet-triplet basis as shown in equation SE6. For a two-proton polaron pair system, there are 4 particles in total (2 polarons and 2 nuclei). The calculation of the four particles based on this singlet-triplet basis can be expressed as the equations given in Equations SE7 and SE8.

$$P = P_e \otimes I_{e2\times2} \otimes I_{e2\times2} = \begin{pmatrix} 1 & 0 & 0 & 0 & 0 & 0 & 0 & 0 \\ 0 & 1 & 0 & 0 & 0 & 0 & 0 & 0 \\ 0 & 0 & 1/\sqrt{2} & 0 & 1/\sqrt{2} & 0 & 0 & 0 \\ 0 & 0 & 0 & 1/\sqrt{2} & 0 & 1/\sqrt{2} & 0 & 0 \\ 0 & 0 & -1/\sqrt{2} & 0 & 1/\sqrt{2} & 0 & 0 & 0 \\ 0 & 0 & 0 & -1/\sqrt{2} & 0 & 1/\sqrt{2} & 0 & 0 \\ 0 & 0 & 0 & 0 & 0 & 0 & 1 & 0 \\ 0 & 0 & 0 & 0 & 0 & 0 & 0 & 1 \end{pmatrix} \otimes I_{e2\times2}. \quad \text{SE7}$$

$$= \begin{pmatrix} 1 & 0 & 0 & 0 & 0 & 0 & 0 & 0 & 0 & 0 & 0 & 0 & 0 & 0 & 0 & 0 \\ 0 & 1 & 0 & 0 & 0 & 0 & 0 & 0 & 0 & 0 & 0 & 0 & 0 & 0 & 0 & 0 \\ 0 & 0 & 1 & 0 & 0 & 0 & 0 & 0 & 0 & 0 & 0 & 0 & 0 & 0 & 0 & 0 \\ 0 & 0 & 0 & 1 & 0 & 0 & 0 & 0 & 0 & 0 & 0 & 0 & 0 & 0 & 0 & 0 \\ 0 & 0 & 0 & 0 & 1/\sqrt{2} & 0 & 0 & 0 & 1/\sqrt{2} & 0 & 0 & 0 & 0 & 0 & 0 & 0 \\ 0 & 0 & 0 & 0 & 0 & 1/\sqrt{2} & 0 & 0 & 0 & 1/\sqrt{2} & 0 & 0 & 0 & 0 & 0 & 0 \\ 0 & 0 & 0 & 0 & 0 & 0 & 1/\sqrt{2} & 0 & 0 & 0 & 1/\sqrt{2} & 0 & 0 & 0 & 0 & 0 \\ 0 & 0 & 0 & 0 & 0 & 0 & 0 & 1/\sqrt{2} & 0 & 0 & 0 & 1/\sqrt{2} & 0 & 0 & 0 & 0 \\ 0 & 0 & 0 & 0 & -1/\sqrt{2} & 0 & 0 & 0 & 1/\sqrt{2} & 0 & 0 & 0 & 0 & 0 & 0 & 0 \\ 0 & 0 & 0 & 0 & 0 & -1/\sqrt{2} & 0 & 0 & 0 & 1/\sqrt{2} & 0 & 0 & 0 & 0 & 0 & 0 \\ 0 & 0 & 0 & 0 & 0 & 0 & -1/\sqrt{2} & 0 & 0 & 0 & 1/\sqrt{2} & 0 & 0 & 0 & 0 & 0 \\ 0 & 0 & 0 & 0 & 0 & 0 & 0 & -1/\sqrt{2} & 0 & 0 & 0 & 1/\sqrt{2} & 0 & 0 & 0 & 0 \\ 0 & 0 & 0 & 0 & 0 & 0 & 0 & 0 & 0 & 0 & 0 & 0 & 1 & 0 & 0 & 0 \\ 0 & 0 & 0 & 0 & 0 & 0 & 0 & 0 & 0 & 0 & 0 & 0 & 0 & 1 & 0 & 0 \\ 0 & 0 & 0 & 0 & 0 & 0 & 0 & 0 & 0 & 0 & 0 & 0 & 0 & 0 & 1 & 0 \\ 0 & 0 & 0 & 0 & 0 & 0 & 0 & 0 & 0 & 0 & 0 & 0 & 0 & 0 & 0 & 1 \end{pmatrix} \quad \text{SE8}$$

The matrix in equation SE7 is a 16 ×16 matrix which represents the 16 quantum states for this four-particle system. The physical meaning of the Kronecker product in equation SE6 is the combinations of the possible quantum states that exist in the four-particle system (or singlet-triplet-proton-proton system).

Therefore, accordingly, the singlet projection operator P_s in equation SE5 can be expressed in Equation SE9:

$$P_s = \frac{1}{4} \times I_{e16\times16} - I_{Ax} \otimes I_{Bx} \otimes I_{2\times2} \otimes I_{2\times2} - I_{Ay} \otimes I_{By} \otimes I_{2\times2} \otimes I_{2\times2} - I_{Az} \otimes I_{Bz} \otimes I_{2\times2} \otimes I_{2\times2}. \quad \text{SE9}$$

Where $I_{e16\times16}$ is a 16 ×16 unity matrix and I_A andI_B, subscript x, y and z, are the corresponding components of the Pauli matrices of each polaron. The effect of the singlet projection operator as used in equation SE5 is to "filter out" all the singlet components among all spin configurations.

Noticeably, the subsequent definition of steady state singlet and triplet yield is different from the previous work and is defined by the new Different-Rate-Polaron-Pair Model. In this model, not only are the two hyperfine fields (two protons) considered in the hyperfine coupling, also included are two different decay pathways for singlet and triplet polaron pair fractions, which contains dissociation and recombination. For different pathways, it is assumed that different rate constants applied to different processes. i.e., $k_{S,d}$, $k_{T,d}$ and $k_{S,r}$, which correspond to the dissociation rates of singlet polaron pair and triplet polaron pair into free charges and the recombination rates of singlet polaron pair into singlet exciton, respectively. Straightforwardly, the steady state yields can be expressed using equations (SE10a), (SE10b) and (SE10c).

$$\Phi_{S,r} = k_{S,r} \cdot \int_0^\infty \rho_S(t) e^{-k_{S,r} \cdot t} dt \qquad \text{SE10a}$$

$$\Phi_{S,d} = k_{S,d} \cdot \int_0^\infty \rho_S(t) e^{-k_{S,d} \cdot t} dt \qquad \text{SE10b}$$

$$\Phi_{T,d} = k_{T,d} \cdot \int_0^\infty \rho_T(t) e^{-k_{T,d} \cdot t} dt \qquad \text{SE10c}$$

where $\Phi_{S,r}$ is the steady state singlet exciton yield due to singlet polaron pair recombination, $\Phi_{S,d}$ is the steady state singlet yield due to singlet polaron pair dissociation, $\Phi_{T,d}$ is the steady state triplet yield due to triplet polaron pair dissociation. $\Phi_{T,d}$, the steady state triplet exciton yield due to triplet polaron pair recombination, is not included as the triplet recombination process can neither contribute to the total current nor the

luminescence of the device. ρ_S is the singlet fraction, and ρ_T is the triplet fraction which can be evaluated using $\rho_S + \rho_T = 1$. In this way the magnetic field dependent yields $\Phi_{S,r}(B)$, $\Phi_{S,d}(B)$ and $\Phi_{T,d}(B)$ are obtained as shown in equation (SE10a), (SE10b) and (SE10c). Electroluminescence is directly related to the radiative recombination of singlet excitons, and magnetoconductance is linked to the dissociation of singlet and triplet polaron pairs as suggested in literature[32]. For the magnetoconductance, the approach by Nguyen and co-workers[32] is used but noticeably, with different rates for dissociation for singlets and triplets in order for the contribution to MC from singlets and triplets to be calculated. This removes the relative weight factor δ_{TS} used in previous work. In this way, intuitively, the expressions for magnetoconductance and magnetoelectroluminescence can be defined as in equations (SE11a) and (SE11b).

$$MEL(B) = \frac{\Phi_{S,r}(B) - \Phi_{S,r}(B=0)}{\Phi_{S,r}(B=0)} \qquad \text{SE11a}$$

$$MC(B) = \frac{|\Phi_{S,d}(B)+\Phi_{T,d}(B)| - |\Phi_{S,d}(B=0)+\Phi_{T,d}(B=0)|}{\Phi_{S,d}(B=0)+\Phi_{T,d}(B=0)} = \frac{\Phi_{S,d}(B)+\Phi_{T,d}(B)}{\Phi_{S,d}(B=0)+\Phi_{T,d}(B=0)} - 1 \qquad \text{SE11b}$$

The expressions for MC and MEL in equations (SE11a) and (SE11b) are the foundation for the newly developed fitting procedure discussed in the main text.

S2. Two proton polaron pair model simulation

In the two proton Polaron Pair model, there are two components of the hyperfine field corresponding to two hyperfine precession frequencies. These two frequencies can be seen in Figure S2(a) which is different to the single oscillation phase caused by a single hyperfine field in our previous work[14]. Under zero external magnetic field and this intertwined oscillation consists of multi-oscillations due to the two different hyperfine precession frequencies. In Figure S2(b), when the external magnetic field is 50μT, which is larger than

the smaller component of the two hyperfine fields (39μT), but smaller than the larger hyperfine component (1.55mT), the oscillation envelope changes and is affected by the Zeeman interaction of the external magnetic field. When the external magnetic field increases to 250μT, which is significantly larger than the smaller hyperfine field component (39μT), as shown in Figure S2(c), the effect of the hyperfine precession of the slow frequency (67.8MHz) is totally eliminated (the envelope in Figure S2 (a) and (b)) and dominated by the much larger effect of the Zeeman interaction with the external magnetic field. However, as the external field is still much smaller than the larger hyperfine field (1.55mT), the high precession frequency is still visible in the oscillation. Similar for Figure S2(d), the only effect is the Zeeman interaction getting more intense as the oscillation envelope becomes faster.

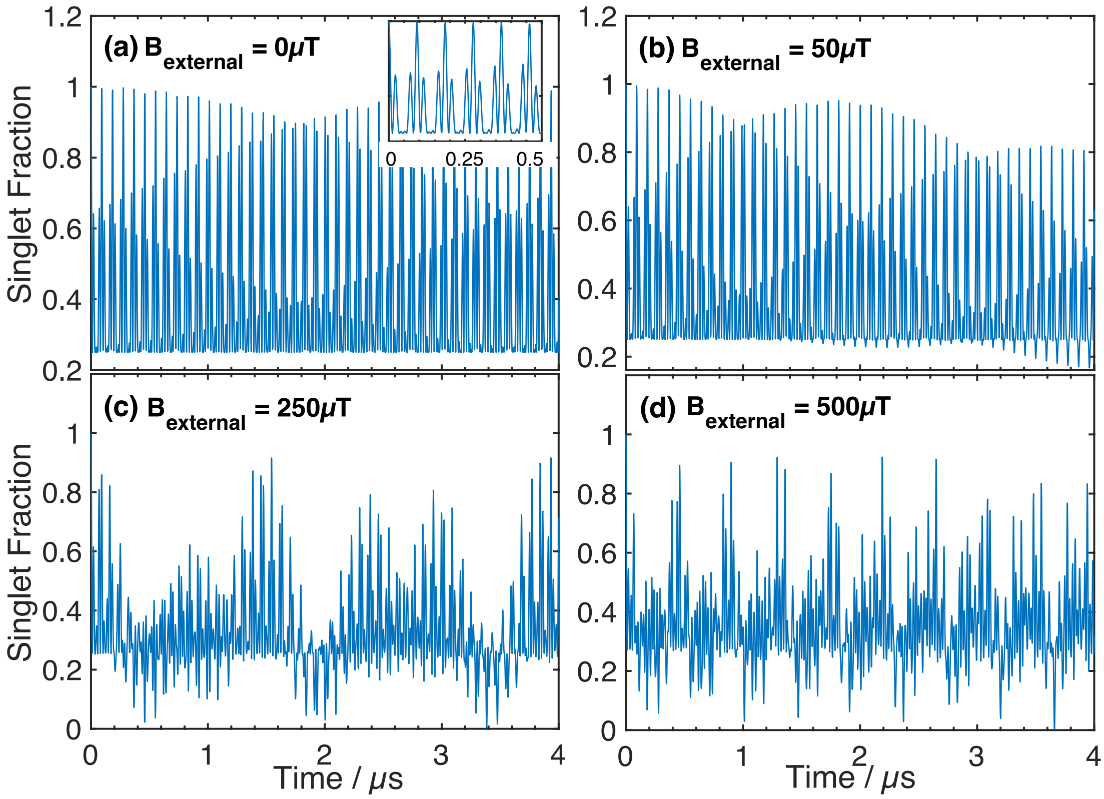

Figure S2: The singlet fraction time evolution under different external magnetic fields using B_{hfc1}= 1.55mT and B_{hfc2}= 0.39mT. The inset in (a) is a detail of the singlet fraction from 0 to 0.5μs.

In this work, the MC and MEL are modelled as in equation SE11 (a) and (b). In Figure S3, the MC and MEL over a large range of magnetic fields ($\pm$20mT) are simulated with model parameters: B_{hfc1} = 1.55mT, B_{hfc2} = 0.39mT, $k_{S,r}$ = 237.7MHz, $k_{S,d}$ = 104.6MHz and $k_{T,d}$ = 96.5MHz. In Figure S3(a), the magnetic field ranges from -20mT to 20mT and the inset is the ultra-small magnetic field region (below 1mT) where the typical "W" shape MC manifests. The simulated MC and MEL using two-proton polaron pair model also tend to saturate at relatively larger magnetic field (approximately larger than |6mT|). This indicates that the polaron pair model is not dominant at high fields, and other HFE starts to play an important role. Evidently, from the HFE measurement experiments in Figure S4 and the current dependence experiments within the ultra-small magnetic field range shown in the

literature[14], it is clear to see that the HFE starts to dominate the MC effect when the external magnetic field exceeds approximately ±30mT. The MC rises rapidly compared to below ±30mT and is orders of magnitude larger than that of the saturated MC level shown in Figure S3(a). This is similar for MEL simulation results in Figure S3(b). Again, the HFE starts to dominate the high fields and the PP model is no longer applicable.

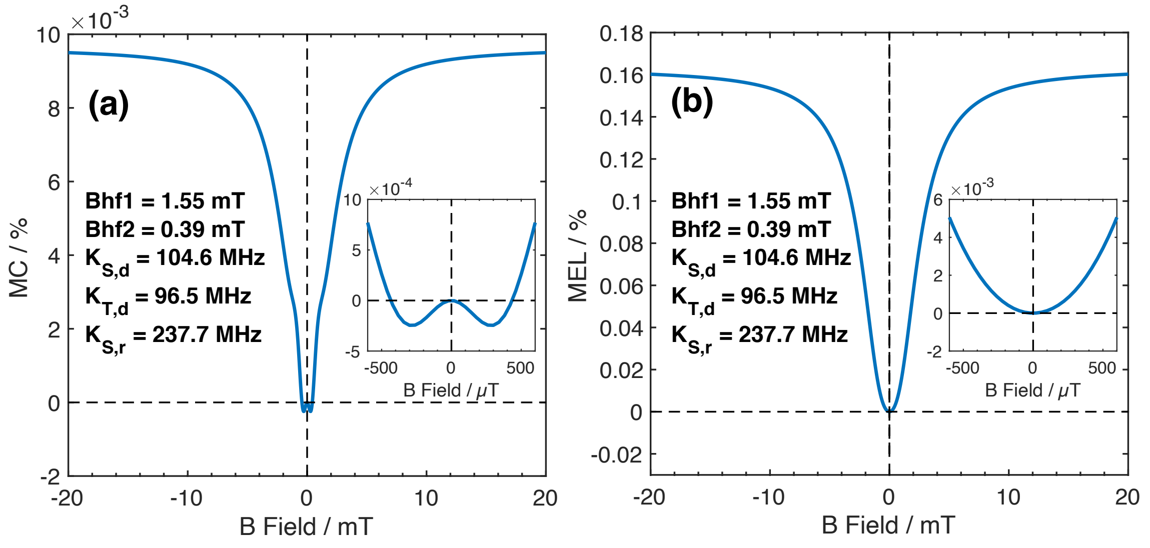

Figure S3: Simulated (a) MC and (b) MEL. The insets in (a) and (b) are USMFE regions of the simulations

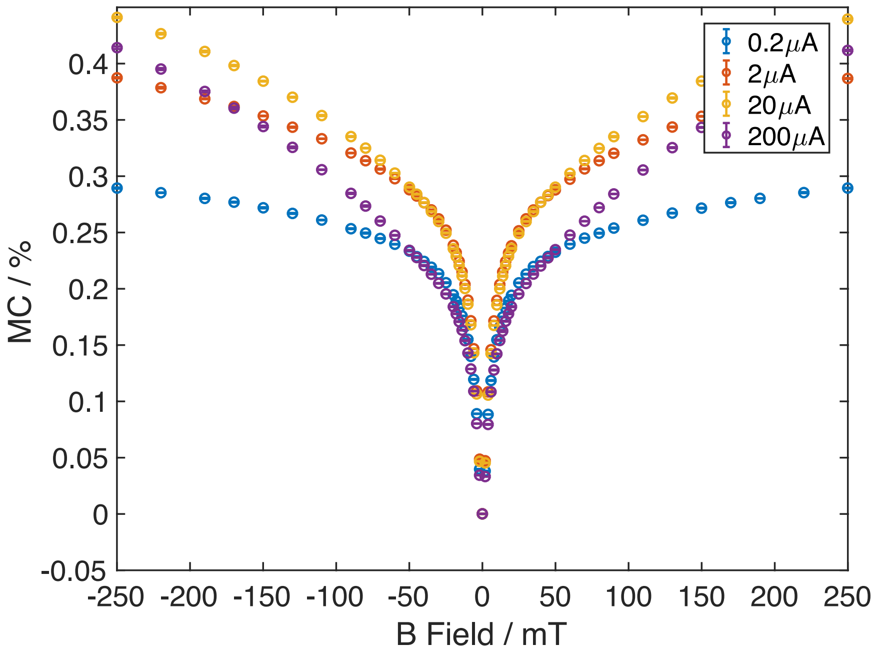

Figure S4: High magnetic field effect MC: drive current dependence measurement

Notably, reference [36] demonstrates that a “small” ratio of the decay rate (recombination or separation) over the hyperfine Larmor frequency can generate a functional “W” shaped MFE, however, it is not clear how “small” is needed to produce the “W” shape MFE. Therefore, in Figure S5, the simulation of changing different rate ratios (singlet or triplet dissociation rate over hyperfine Larmor frequency) was carried out. Evidently, the “W” shaped MC starts to disappear when the rate ratio is above approximately 0.57. Therefore, when the rate ratio is below 0.57, the value can be considered as “small” enough to generate the functional “W” shaped MC in our model.

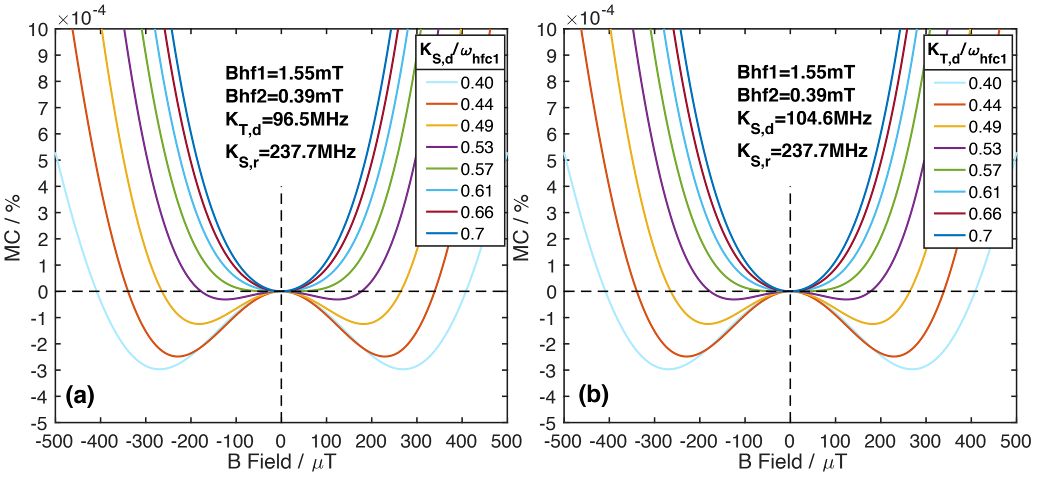

Figure S5: Simulations on MC form changes upon different ratios of (a) singlet dissociation rate over the hyperfine frequency (${k_{S,d}}/{\omega_{hfc1}}$) and (b) triplet dissociation rate over the hyperfine frequency (${k_{T,d}}/{\omega_{hfc1}}$).

Additionally, as we saw no “W” shaped behaviour in the MEL we simulated the effect of the hyperfine field on the singlet recombination. In order to observe a “W” effect (shown in Figure S6) it is necessary to change the ratio of the singlet recombination rate over the hyperfine Larmor frequency (${k_{S,r}}/{\omega_{hfc1}}$). For a given singlet recombination rate ($k_{S,r} = 88MHz$) we can adjust the hyperfine field to see its effect on the MEL. In Figure S6 it can be seen that the “W” shape in the MEL only starts to appear when one of the hyperfine fields is greater than 0.98mT which corresponds to a ${k_{S,r}}/{\omega_{hfc1}}$ value of 0.51. Therefore, the lack of a “W” feature in the MEL is a clear indicator of the upper limit of the hyperfine fields present.

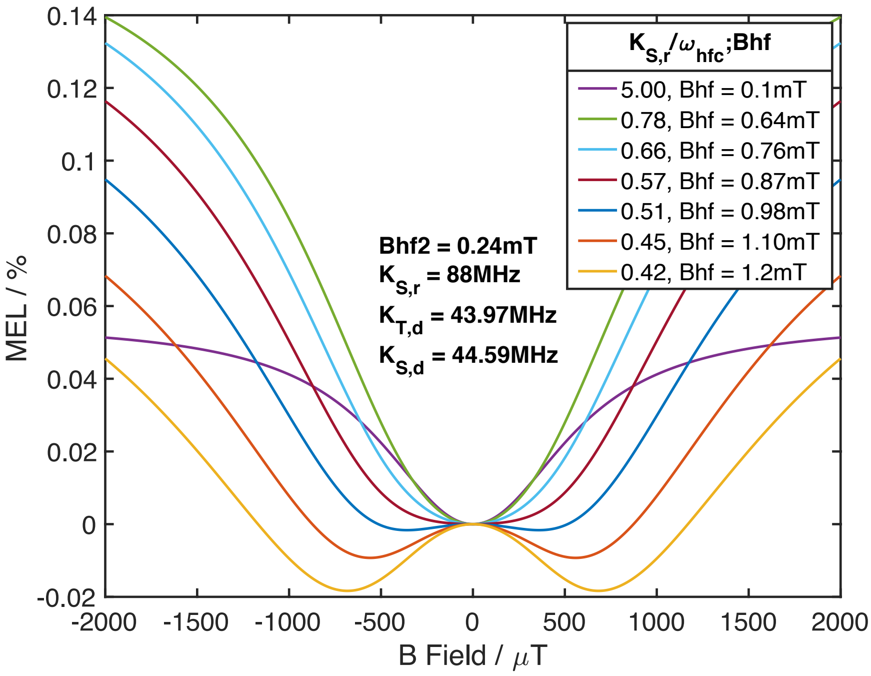

Figure S6: Simulations on MEL form changes upon different ratios of Alq_3 singlet recombination rate over the hyperfine frequency ($^{k_{S,r}}/_{\omega_{hfc1}}$)

S3. Approximation of Average Local Hyperfine Fields for HOMO and LUMO in Alq_3 From DFT calculations

Whilst the two-proton model describes the interaction of an electron or hole with two isolated hydrogen nuclei in reality the electron and hole are actually experiencing a local hyperfine environment from the various nearby atoms. In order to estimate the effective local hyperfine field we use literature values[53] calculated using Density Functional Theory (DFT) for both the Lowest Unoccupied Molecular Orbital (LUMO) and Highest Occupied Molecular Orbital (HOMO).

In order to carry out the approximation and to describe the Alq_3 molecule using the same referencing number system, the atomic numbering sequence in reference[53] is applied to all the Alq_3 molecules described in references[46-51]. From reference[53], the calculated local

hyperfine fields of an anion Alq_3 based on DFT are shown in Table ST1. There are three columns of hyperfine field information, corresponding to the x, y and z components of the anisotropic hyperfine field of a given atom.

Atom	hfc (mT)		
	X / mT	Y / mT	Z / mT
**Al**	-0.189	-0.172	-0.116
**N1**	0.047	0.053	0.095
**H1**	-0.059	-0.044	0.066
**H2**	-0.014	-0.008	0.019
**H3**	-0.067	-0.048	-0.011
**H4**	-0.01	-0.009	0.007
**H5**	-0.006	-0.006	0.008
**H6**	-0.01	-0.006	0.012
**N2**	0.057	0.064	0.739
**H7**	-0.666	-0.496	-0.201
**H8**	-0.01	0.07	0.119
**H9**	-1.152	-0.781	-0.336
**H10**	-0.171	-0.165	-0.039
**H11**	-0.093	-0.08	0.009
**H12**	-0.093	-0.08	-0.001
**N3**	0.045	0.054	0.453
**H13**	-0.418	-0.306	-0.136
**H14**	-0.006	0.04	0.081
**H15**	-0.731	-0.497	-0.213
**H16**	-0.108	-0.103	-0.02
**H17**	-0.059	-0.05	0.009
**H18**	-0.059	-0.041	-0.001

Table ST1. DFT calculated local hyperfine fields of an anion Alq_3. Data reproduced from reference[53].

After obtaining information on the local hyperfine fields, the local hyperfine fields can be approximated through an average of the hyperfine fields at given atom's position. The averaging method is presented in references[24,52] and can be expressed as in equation SE12.

$$< a >= \sqrt{\sum_k a_k^2 I_k (I_k + 1)} \qquad \text{SE12}$$

Where $< a >$ is the average local hyperfine field, a_k is the total local hyperfine field for the specific atom (or hyperfine coupling constant), I_k is the spin quantum number of that atom.

A specific example for the LUMO in Figure 2(c) in Reference [51] is presented for clarification. As can be seen, the LUMO spatial distribution in Figure 2(c) in Reference [51] is indicated by the probability clouds according to the DFT calculation. The LUMO occupied regions include the following atoms: N2, H7, H8, H9, H10, H11, H12, N3, H13, H15. Using the labelling described in Figure 1 in Reference [53], Table ST2 is constructed using X, Y and Z components of the hyperfine field given in reference[29] (also in Table ST2). The resultant total field (or a_k) is calculated using $a_k = \sqrt{X^2 + Y^2 + Z^2}$. I_{spin} is the spin quantum number quantum number for each atom and B1 is evaluated using $a_k^2 I_{spin}(I_{spin} + 1)$ as shown in equation SE12.

	X / mT	Y / mT	Z / mT	a_k	I_{spin}	B1
**N2**	0.057	0.064	0.739	0.744	1	1.10
**H7**	-0.666	-0.496	-0.201	0.854	0.5	0.55
**H8**	-0.01	0.07	0.119	0.138	0.5	0.01
**H9**	-1.152	-0.781	-0.336	1.432	0.5	1.54
**H10**	-0.171	-0.165	-0.039	0.241	0.5	0.04
**H11**	-0.093	-0.08	0.009	0.123	0.5	0.01
**H12**	-0.093	-0.08	-0.001	0.123	0.5	0.01
**N3**	0.045	0.054	0.453	0.458	1	0.42
**H13**	-0.418	-0.306	-0.136	0.54	0.5	0.22
**H15**	-0.731	-0.497	-0.213	0.91	0.5	0.62

Table ST2: An example calculation of the average local hyperfine field of the LUMO of Alq_3

Lastly, the $< a >$ of the whole LUMO region can be calculated as in equation SE13, yielding an average hyperfine field for the LUMO region of 2.13mT.

$$< a > = \sqrt{\sum_{all\ LUMO\ covered\ atoms} B1(n)} = \sqrt{1.1 + 0.55 + \cdots + 0.62} \approx 2.13mT \quad \text{SE13}$$

Hence, the averaged local LUMO hyperfine field is approximately 2.13mT using the data in reference[51].

In order to compare the calculated LUMO and HOMO regions in a single Alq_3 molecule, atoms corresponding to HOMO and LUMO regions from different works are illustrated in Figure S7[46-51], respectively. The bar chart in Figure S7 shows occurrences of different atoms in Alq_3 in LUMO or HOMO from the six different papers. The occupied atoms in LUMO or HOMO are also highlighted in Figure S7 as indicated. It is clear that the HOMO and LUMO spatial distributions is clearly separated from the DFT computations from the literature. It is this spatial separation of HOMO and LUMO, where the holes and electrons reside in the molecule, that makes the local hyperfine fields for electrons and holes different.

As can be seen from the bar charts in Figure S7, different references yield different distributions for HOMO and LUMO regions. In order to include all the possibilities in the

calculations, all atoms corresponding to either HOMO or LUMO in all references are included in the sum of Equation SE13. Therefore, for the LUMO, atoms N2, H7, H8, H9, H10, H11, H12, H13, H14, H15 and H16 are included. For HOMO, atoms N1, H1, H2, H3, H4, H5, H6, H10, H11, H12, H16, H17 and H18 are included.

Similarly, using this approximation method, the average local hyperfine fields are found to be $< a_{LUMO} > \approx$ 2.13 mT and $< a_{HOMO} > \approx$ 0.36 mT. These two different numerical values suggest the different local hyperfine environments for electrons and holes. Although these are only approximations, errors can arise in many ways, including the different DFT calculation processes and methods used by different authors. The averaging formula in equation SE12 is itself an approximation and identifying individual atoms for LUMO and HOMO in different papers can be hard to determine. Additionally, the sum in Equation SE13 has no weighting corresponding to the HOMO and LUMO probability density.

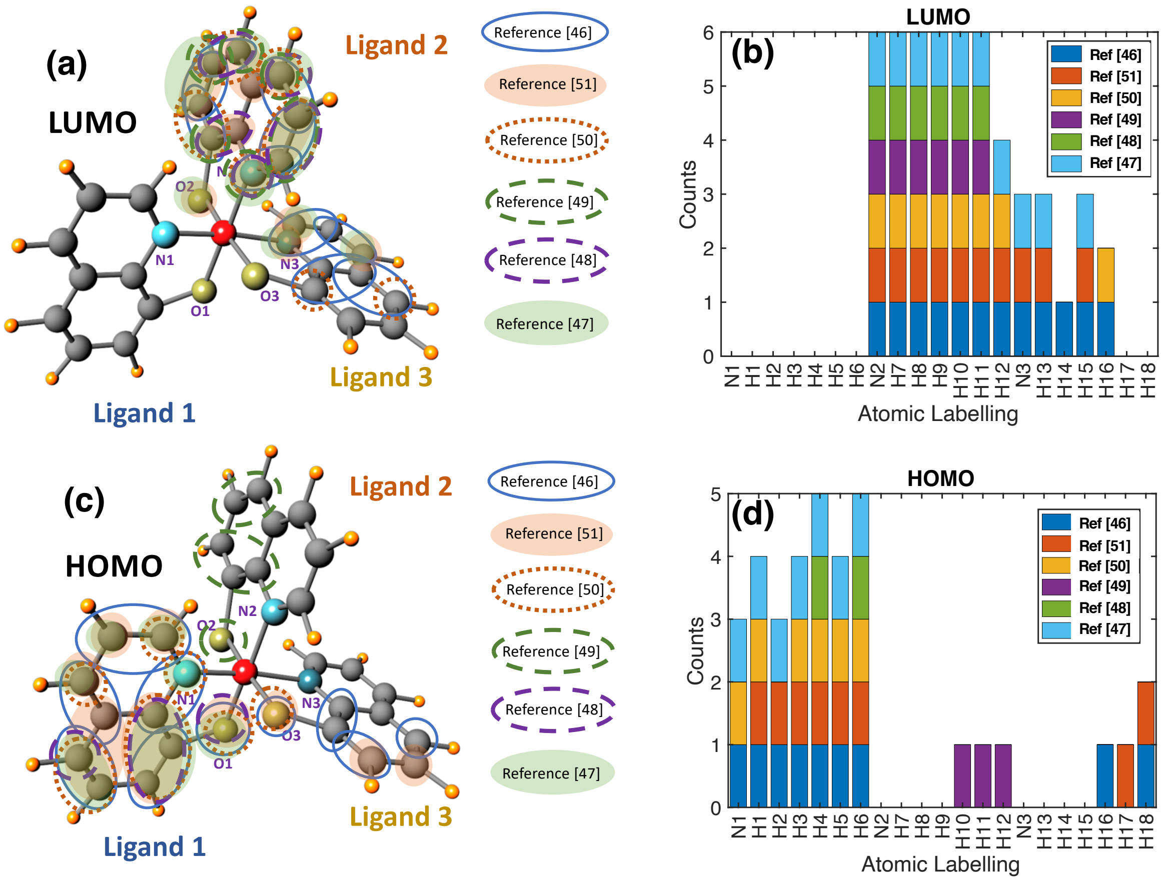

Figure S7. Spatial distribution of (a) LUMO and (c) HOMO in Alq_3 using different literature sources[46-51]. (b) and (d) show the stats of atomic occurrences in different sources[46-51].

In order to compare the simulation results using the fitting-extracted local hyperfine fields (Figure 3) and the DFT-estimated local hyperfine fields, a simulation was carried out and the results are shown in Figure S8. In the simulation, Figure S8 (a), all the rates (singlet and triplet polaron pair dissociation rates $k_{S,s}$, $k_{T,s}$ and singlet polaron pair recombination rate $k_{S,r}$) are set as: $k_{S,s} = 44.59MHz$, $k_{T,s} = 43.97MHz$ and $k_{S,r} = 88MHz$. As indicated in the main text, when $B_{hf1} = 0.63mT$, $B_{hf2} = 0.24mT$, the MC displays a clear "W" shape. However, when the DFT-estimated hyperfine fields are fed into the simulation (using the same rates), the "W" shaped MC disappears as shown in Figure S8 (a). If we run a simulation where we force the DFT-estimated hyperfine fields and allow free fitting of the rates we can

obtain a "W" shaped MC, shown in Figure S8 (b) but it can be seen that the minima have moved to much larger fields and do not match the experimental data.

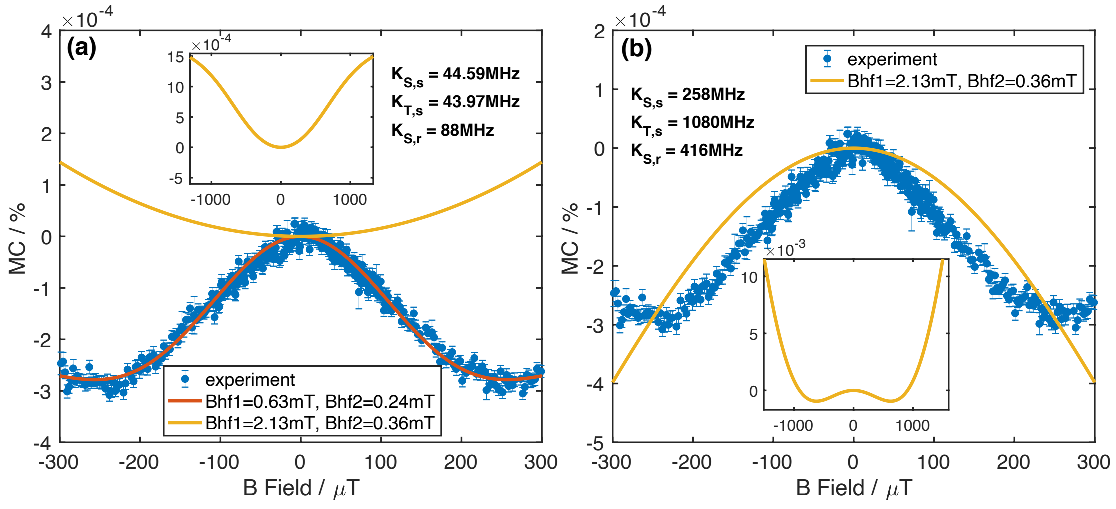

Figure S8. Simulations of MCs by fixing the local hyperfine fields with the estimated DFT values (a) while keeping the dynamics rates the same as the experimental data extracted rates (in the main text) (b) while fitting the dynamics rates to the experimental data.

S4. Reproducibility checks

Reproducibility checks were performed to ensure both the working device performance and the two-proton polaron pair model fitting procedure are valid. First, the current dependence of the MC and MEL on the device is carried out with different device drive currents (39μA, 76μA and 113μA). Those data are then fitted using the two-proton polaron pair model. The fitting results for different drive current are shown in Table ST3.

Drive Current	B_{hfc1} / mT	B_{hfc2} / mT	$k_{S,\mathrm{r}}$ / MHz	$k_{S,d}$ /MHz	$k_{T,\mathrm{d}}$ / MHz
**39 μA (main text)**	0.63±0.01	0.24±0.01	87.97±5.56	44.59±0.01	43.97±0.01
**76 μA**	0.66±0.01	0.24±0.01	92.37±8.73	46.82±0.16	46.16±0.15
**113 μA**	0.66±0.02	0.22±0.02	95.15±5.77	46.80±0.14	46.23±0.14

Table ST3 Fitting parameters for different drive currents of the same device within $\pm300\mu$T

From Table ST3, all the fitted parameters (B_{hfc1}, B_{hfc2}, $k_{S,d}$, $k_{T,d}$ and $k_{S,r}$) are consistent within errors in the same device within the magnetic field range of $\pm300\mu$T. The larger uncertainties in the values of $k_{T,d}$ and $k_{S,r}$ at higher currents are due to the lower number of repetitions in those experiments. The MC is averaged over 800 times at a drive current of 39 μA while the others are only averaged for around 100 times (76μA and 113μA). The consistency in the data fitting results in Table ST3 suggests that it is valid to fit both MC and MEL below $\pm300\mu$T.

Device	B_{hfc1} / mT	B_{hfc2} / mT	$k_{S,\mathrm{r}}$ / MHz	$k_{S,d}$ /MHz	$k_{T,\mathrm{d}}$ / MHz
**Device I (main text)**	0.63±0.01	0.24±0.01	87.97±5.56	44.59±0.01	43.97±0.01
**Device II**	0.64±0.01	0.23±0.01	90.69±8.73	45.30±0.16	44.82±0.15
**Device III**	0.64±0.02	0.24±0.02	89.47±5.77	45.17±0.14	44.69±0.14

Table ST4 Fitting parameters for different devices under the drive current of 39μA within $\pm300\mu$T

In order to check if the device performance can be reproducible, three different devices were fabricated, and the same measurement of the MC and MEL conducted under a drive current of 39μA has been carried out. The results of this reproducibility test are shown in Figure S9. It is noticeable that the noise level in Figure S9 (c), (d), (e) and (f) are much larger than in (a) and (b). This is due to large number of averages in (a) and (b) (more than 800 repetitions) compared to (c), (d), (e) and (f) (only 100 repetitions). Despite the noise level, the dip position in the MC and the overall magnitudes of the MC and MEL are consistent across different devices as can be seen from Figure S9. The same fitting process is carried

out on each of the data sets and the results are shown in Table ST4. All the fitting parameters are significantly and statistically consistent across devices.

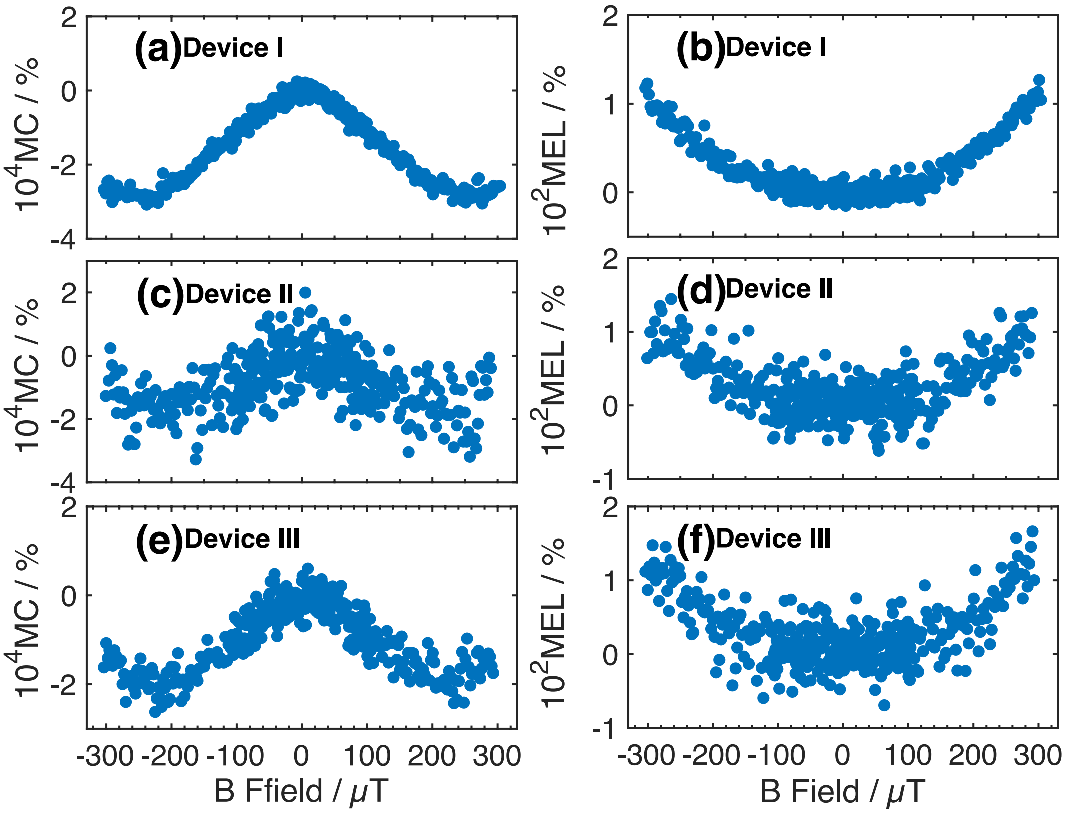

Figure S9. The MC and MEL within $\pm 300\mu T$ at a drive current of $39\mu A$ for three different devices

Additionally, in order to check if there's any significant device degradation occurring during this long time of MC and MEL measurement, a degradation check is carried out as shown in Figure S10. More than 950 measurements were taken on a single device, which means that the device was continuously working for approximately 20 days. Data were collected and averaged using 4 approaches, as shown in Figure S10: First 100 measurements, mid 300 – 700 measurements, last 900 – 966 measurements and all 966 measurements. Notably, the first 100 measurements averaging are noisy, however, the trend (either MC or MEL) is following the route of the all 966 averaged data. For the last 900 – 966 measurements, of which the device has already worked for over 18 days, the averaged MC and MEL are still

following the exact route of the all 966 averaged data. It means that the device under test hasn't gone through any significant degradation during measurement, and there is no effect on the result of MC and MEL data. The consistency in the fitting results on different averaging numbers further justifies the non-degradation of the device and the results are shown in Table ST5.

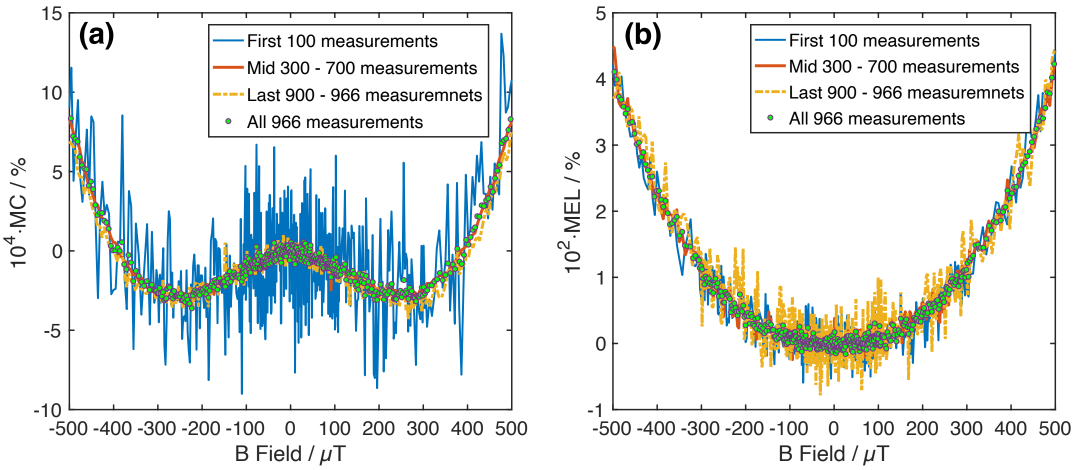

Figure S10 Degradation check on the OLED (a) MC (b) MEL

Averages	B_{hfc1} / mT	B_{hfc2} / mT	$k_{S,r}$ / MHz	$k_{S,d}$ /MHz	$k_{T,d}$ / MHz
**First 100**	0.63$\pm$0.03	0.24$\pm$0.04	89.83$\pm$14.52	44.90$\pm$0.34	44.31$\pm$0.28
**Mid 300~700**	0.63$\pm$0.01	0.24$\pm$0.01	88.64$\pm$6.63	44.37$\pm$0.02	43.76$\pm$0.02
**Last 900~966**	0.62$\pm$0.02	0.24$\pm$0.01	87.35$\pm$14.74	43.87$\pm$0.04	43.25$\pm$0.04
**All 966**	0.63$\pm$0.01	0.24$\pm$0.01	87.97$\pm$5.56	44.59$\pm$0.01	43.97$\pm$0.01

Table ST5 Fitting parameters for different averages in Figure S10 under the drive current of 39μA within $\pm$300μT

S5 Experimental set-up and device structure

The system consists of 3 pairs of orthogonal coils of different sizes oriented in the x, y and z directions (referred to as x, y and z coil pairs). The sample was mounted in a sample holder, located at the center of the system. The homogeneity of the magnetic field generated by the coil across the device was measured and is shown in Figure S11.

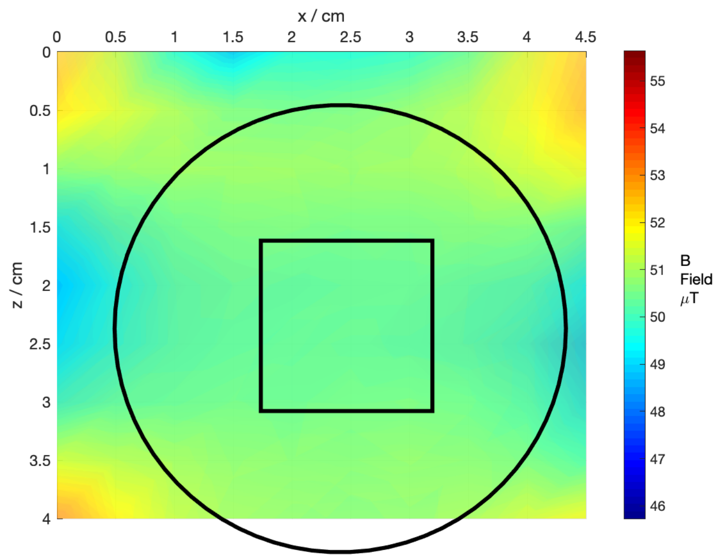

Figure S11. Homogeneity of the magnetic field along XZ plane.

In Figure S11, the circle region is where the device holder is and the 1.5 x 1.5 cm square region is where our device lies. The actual diode is a 2 x 2 mm region within this square. In order to measure this homogeneity, the gaussmeter was first placed in the centre of the square and the Z coil current adjusted to produce a B field of 51 μT at that point. With the coil current fixed we moved the gaussmeter to pre-mapped coordinates to map the magnetic field across the region. The average B field across the device is 51.1μT$\pm$0.2μT.

The whole coil setup and the device were located in a position where the Earth magnetic field components along the x, y and z directions were measured to be approximately 14μT, 42μT and 10μT respectively. The magnetic field along the z direction was chosen as the

scanning B field and the x and y applied DC fields simply used to cancel the Earth's field components. In this example, the x and y coils were connected to a DC power supply (Siglent SPD3303X) while the z coils were connected to a source measure unit (Keithley 2400 SourceMeter).

The sample holder is designed with no ferromagnetic components (for example, with custom made phosphor-bronze springs in the sample contacts) which could provide stray fields or result in sample movement as the external field is switched.

S6 Data mean values and error bars.

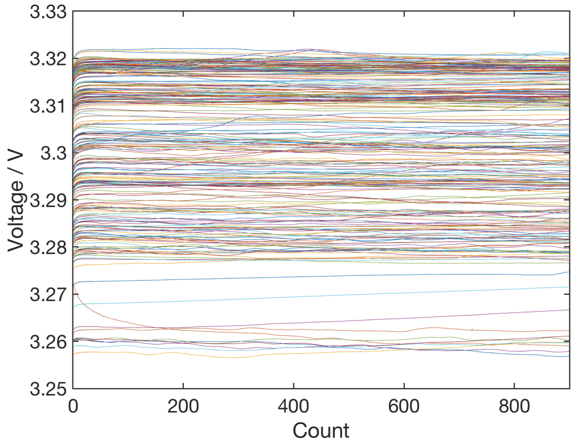

Figure S12. Constant current mode raw data of device voltage versus count. 100 repetitions of the acquisition are displayed under 2μA.

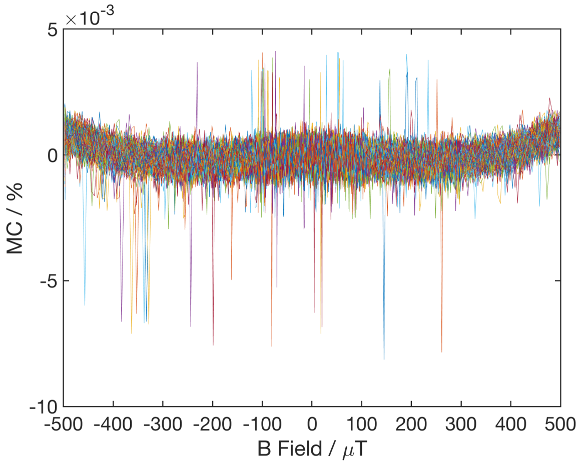

Figure S13. MC values calculated using equation (1a) for each of the 100 repeated acquisitions.

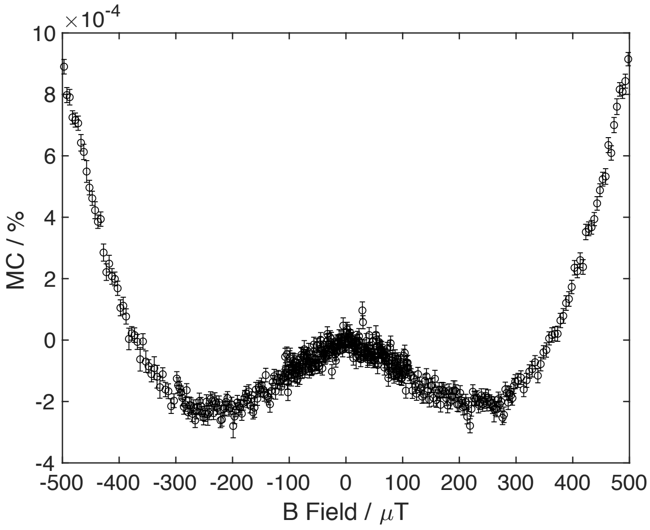

Figure S14. Resultant averaged MC, including the standard errors from 100 MC acquisitions.

The MC was measured when the diode was biased with constant current and the device voltage was recorded at each applied magnetic field. This is an example of how our MC (and MEL) data is averaged. Figure S12 shows the raw data of device voltage plotted against the number of counts. The MC was measured under constant drive current and Figure S12 represents the measured device voltage versus count, where the count represents any applied magnetic field and is always alternated with a null field measurement. Thus, the

very small device drift, < 2% over the whole measurement time, evident in Figure S12 is eliminated from the MC calculation. Equation (1a) was applied to calculate each MC value for the 100 repeated datasets (raw MC data is shown in Figure S13). The arithmetic mean and standard error were calculated from these 100 datasets using equations (SE14) and (SE15):

$$MC_{mean}(B) = \frac{1}{n}\sum_{n=1}^{100} MC_n(B) \qquad \text{SE14}$$

$$Standard\ Err. = \frac{\sigma}{\sqrt{n}} \qquad \text{SE15}$$

where $MC_n(B)$ is the MC value of the n-th repeated dataset at a specific applied magnetic field and σ is the standard deviation of each data point from the 100 experiments. The calculated MC_{mean} and $Standard\ Err.$ correspond to the MC values and error bars shown in Figure S14. Likewise, the MEL is averaged in the similar way as discussed above.

S7 Model optimization and fitting

The data-model optimization and fitting process is are realized by minimizing a global reduced χ^2 as defined in equation SE16.

$$\chi^2_{global,red} = \sum_N \left[\left(\frac{MC_{exp}-MC_{model}}{MCerror_{exp}}\right)^2 + \left(\frac{MEL_{exp}-MEL_{model}}{MELerror_{exp}}\right)^2\right]. \\ = \chi^2_{red,MC} + \chi^2_{red,MEL} \qquad \text{SE16}$$

Where MC_{exp}, MEL_{exp}, $MCerror_{exp}$ and $MELerror_{exp}$ are the experimentally obtained MC, MEL and their corresponding standard errors respectively. MC_{model} and MEL_{model} are the simulated data produced from Eqs (4a) and (4b). The minimization is processed using Nelder-Mead Method and achieved in MATLAB.

References

S1. Hayashi, H. *Introduction to Dynamic Spin Chemistry*. (World Scientific Publishing Co. Pte. Ltd., 2004).
